# Results of Noncontrast Brain Computed Tomography Scans of 1-18 Year Old Epileptic Children

**Published:** 2012

**Authors:** Razieh FALLAH, Reza NAFISI MOGHADAM, Mohammad FALLAH TAFTI, Mahdi SALMANI NODOUSHAN

**Affiliations:** 1 Associate Professor of Pediatric Neurology, Department of Pediatrics, Shahid Sadoughi University of Medical Sciences, Yazd, Iran; 2 Assistant Professor of Radiology, Department of Radiology, Shahid Sadoughi University of Medical Sciences, Yazd, Iran; 3 Medical Student, Ali Ben Abitalib Medical College, Islamic Azad University of Yazd, Yazd, Iran

**Keywords:** Epilepsy, Brain CT scan, Children, Neuroimaging

## Abstract

**Objective:**

The advent of computed tomography (CT) scan revolutionized the diagnostic evaluation of neurologic patients. The aim of this study was to evaluate brain CT results of epileptic children.

**Materials & Methods:**

In a descriptive cross-sectional study, noncontrast brain CT scan of 150 consecutive 1-18 year old epileptic children whom were referred to pediatric neurology clinic of Shahid Sadoughi University of Medical Sciences, from May 2008 to October 2010 in Yazd-Iran, evaluated.

**Results:**

Sixty two girls and 88 boys with mean age of 6.6 ± 4.3 years were evaluated. In 38 (25.3 %) children, seizure onset age was under one year and 38 others had abnormal mental / developmental status. Fifty three children (35.3 %) and 97 (64.7%) had partial and generalized seizures, respectively. Partial seizures were more prevalent in children with seizure onset in < 1 year [41.5% (22/53) vs. 16.5% (16/97)] Result of CT was normal in 74 % (n=111). Among the patients with abnormal results, 18(46%) had brain atrophy, 10 (25.6%) structural CNS dysgenesia, six (15.4%) intracranial calcification, three (7.8%) hydrocephaly and two had (5.2%) brain tumor.

Abnormal brain CT was more prevalent in patients with seizure onset in less than one year of age [60.5% (23 of 38) vs. 14.3% (16 of 112), p = 0.003], partial epilepsy [51% (27 of 53) vs. 12% (12/97)], and abnormal developmental status [ 81.5% (31 of 38) vs.7% (8 of 112]. Mean age of seizure onset in epileptic children with abnormal brain CT scan was less (M ± SD:1/17 ± 0.6 years versus 4.02±1.9 years).

**Conclusion:**

Brain CT scan might be considered in evaluation of epileptic children with partial seizures, seizure onset in less than one year of age or neurodevelopmental delay.

## Introduction

Up to five percent of general population suffers from one nonfebrile seizure during his lifetime (1). The prevalence of epilepsy which is defined as two or more unprovoked seizures in intervals of more than 24 hours, is 4.3 to 9.3 in 1000 children (2).

The advent of brain computed tomography (CT) scan revolutionized the diagnostic evaluation of neurologic patients. Neuroimaging studies are valuable in diagnosis of etiology, management and predicting of prognosis in children with epilepsy (3). 

In new onset seizure, emergency neuroimaging is indicated in patients with focal neurological signs, disturbance in the level of consciousness, presence of trauma, intracranial hypertension and in the suspected to vascular or infectious etiology based on clinical and biological data(4).

“ Based on the guidelines of the American Academy of Neurology, in evaluation of first afebrile seizure in children, urgent neuroimaging must be done in seizure with postictal focal neurologic deficits or in children who do not return to baseline neurologic function within several hours and non urgent neuroimaging is recommended in certain clinical situations such as : cognitive or motor impairment of uncertain etiology, unexplained abnormalities on neurologic examination, abnormal electroencephalography not representing a benign syndrome, seizures of partial onset, or in children under the age of one year” (5). 

Magnetic resonance imaging (MRI) is the choice technique for investigation of epileptic patients but CT scan is usually more accessible in emergency conditions to diagnose etiology of seizure and it is useful when MRI may not be readily available. In absence of MRI, CT scanning can show gross structural pathology (6) and it may have a significant role in therapeutic strategy and may postpone MRI investigation. No imaging is needed in simple febrile seizures (4).

In newborns, CT scan or MRI must be done in case of seizures or loss of consciousness to exclude ischemic, traumatic or infectious lesions (7). 

Although neuroimaging is mostly used in evaluation of seizures and epilepsy. But, there is little information about its effectiveness in our country and our city as well. So, the purpose of this study was to evaluate brain CT scan results of epileptic children in Yazd, central city of Iran.

## Materials & Methods

In a descriptive cross-sectional, noncontrast brain CT scans of consecutive epileptic children whom were referred to pediatric neurology clinic of Shahid Sadoughi University of Medical Sciences, from May 2008 to October 2010 in Yazd-Iran, evaluated.

Sample size based on Z formula and confidence interval of 95% with 80% power to detect a significant difference of 0.05, was assessed in 150 patients.

In this study, the diagnostic criteria for epilepsy were based on International League Against Epilepsy (ILAE) classification (8).

In research period, MRI in research period was not available in our center and we had to use CT scan as a neuroimaging diagnostic tool whenever neuroimaging had indication in epileptic children, based on the decision of clinical neurologist. If CT scan was normal and based on clinical decision, MRI should be done (for example in partial epilepsy), the child would be referred to MRI center of another hospital. 

Care was taken to include:

1- Epileptic patients aged 1 -18 years 

2- With no history of major head trauma (as they might have abnormal CT scans)

3- Without febrile seizure

4- Those whose CT scan was done in one center (CT unit of Shahid Sadoughi Hospital) CT machine Characteristic in our hospital was multi slice scanner, by the model of Somatom Spirit and manufactured by the Siemens in 2007.

Assessment and selection of the patients, history taking, physical examination, evaluation of mental/ developmental status of them and identification of epilepsy and seizure type was done by a clinical pediatric neurologist. Seizures with clear partial features by history and direct interview with children parents, observation, examination or EEG findings, were classified as partial. Noncontrast brain CT scans of these children were interpreted by one pediatric neurologist and two radiologists. 

Variables such age, sex, age of seizure onset, seizure type, mental or developmental status, and brain CT scan results, were reviewed.

Chi-square test was used for data analysis of qualitative variables and mean values were compared using independent T-test. Differences were considered significant at P values of less than 0.05. This study has been approved by the ethic committee of Shahid Sadoughi University of Medical Sciences, Yazd, Iran.

## Results

Sixty two girls (41.3%) and 88 boys (58.7%) with mean age of 6.6 ± 4.3 years (range = 1-18 years) were evaluated.

Onset age of seizures was from one months to 17 years (mean ± SD: 3.15 ± 2. 6 years) and in 38 children, seizure onset age was under one year. 

From the viewpoint of seizure type, 53 children (35.3 %) had partial seizures. In 97 patients with generalized seizures, 57(58.8%) had generalized tonic-clonic, 14 (14.4%) absence, 12 (12.4%) myoclonic, eight (8.2%) tonic and six (6.2%) atonic seizures.

Thirty eight (25.3%) of patients had abnormal mental/ developmental status among whom 22 had partial seizures and seizure onset age was under one year in 11 of them. 

Table 1 shows frequency distribution of abnormal developmental status and partial seizure based on seizure onset age which indicates that partial seizures were more prevalent in children with seizure onset in less than one year of age but frequency of developmental delay was not statistically different in children with seizure onset <1 year of age or ≥ one year old. 

The brain scan was normal in 74% of children (n=111). In patients with abnormal results, 18(46%) had brain atrophy or decreased volume of brain, 10 (25.6%) structural CNS dysgenesia (porencephaly, polymicrogyrias, lissencephaly,…) six (15.4%) intracranial calcification, three (7.8%) hydrocephaly and two (5.2%) brain tumor. 

Abnormal brain CT was statistically more prevalent in children with seizure onset in less than one year of age [60.5% (n= 23 of 38 children) of patients with seizure onset under one year of age vs. 14.3% (16 /112) with seizure onset ≥ 1 year of age, p = 0.003], partial seizure [51% (27/ 53) of children with partial seizure vs. 12% (12/97) with generalized seizure and p = 0.02] and abnormal mental/ developmental status [ 81.5% (31 /38) of patients with abnormal developmental status vs.7% (8 /112) with normal developmental status, p=0.001]. 

Brain CT scan results based on sex distribution of patients mean of age and mean of age of seizure onset is illustrated in Table 2 which indicates that frequency of abnormal CT scan result was not statistically different in both sexes and age of seizure onset in epileptic children with abnormal brain CT scan was less.

Comparison of seizure onset age, developmental status and brain CT scan results based on seizure type are presented in Table 3 which shows that seizure onset in less than one year of age, developmental delay, and abnormal brain CT scan were more prevalent in children with partial seizures.

## Discussion

Majority of published articles have evaluated pediatric neuroimaging of the first afebrile seizure, while in this study, brain CT scan of 1-18 year old epileptic children was evaluated.

In present study, boys were more than girls (58.7% vs. 41.3%) and it is in agreement to another study in USA (52% boys vs. 48% girls) (9).

Ten percent of children with first afebrile seizures in another Iranian study (10), 12.7% in Berg study (11), 21.2 % in Maytal study (12), 35.2% in Hsieh et

al study (9), 49% in Indian study (13), 50% in a study in Mexico (14), 51.5 % (15) and 52.6% (16) in two studies in Nigeria, had abnormal brain CT scan, while in present study, brain CT scan was abnormal in 26 % of epileptic children. Possible explanations for these discrepancies are differences in: sample size, age of patients, geographic area and method of patient selection (first seizure or new onset epilepsy). 

In a study in Nigeria, hydrocephalus was the most common finding in CT scan of 103 children with seizures disorders (15) and in another Nigerian study, cerebral infarct and cerebral atrophy were the most common lesions of brain CT in 19 infants (16). In Indian study, the most common CT abnormality in 162 one month – 12 year old children with generalized epilepsy was a ring/disc like enhancing lesion (13) and in Mexican study, cerebral atrophy and neurocysticercosis were the most frequent abnormalities in 118 children with epilepsy (14) and in Hsieh et al study, cerebral dysgenesis was the most common abnormal finding in less than two year old children (9). But, in our study, brain atrophy and CNS structural dysgenesis were more prevalent abnormalities of brain CT scan.

In present study, abnormal brain CT scan was more prevalent in patients with partial seizures which is consistent with other studies (9, 10, 12-15).

In our study, abnormal neuroimaging result was more frequent in patients with mental- developmental deficit which supports other studies (9, 10).

In Hsieh et al study which evaluated neuroimagings of less than two year old infants with new onset afebrile seizures, about 50% of less than one year old children had partial seizure (9). However, in present study, 57.9 % of children with seizure onset in less than one year of age, had partial seizure. One possible explanation for this mild discrepancy is difference in the age of patients.

In this study, children with seizure onset in infancy (less than one year) had more partial seizures and it is in agreement to other studies (17, 18). Since infants are more likely to have a localization-related epilepsy (17) and focal malformation of cortical development (18) which may present as partial seizures, this result supports the recommendation of the literature that neuroimaging should be done in first afebrile seizure in children under the age of one year (5). Accessibility of imaging modality is one of important factors of selection of choice imaging modalities and since CT is more widely available, it can be a useful neuroimaging tool, especially in urgent situations (9).

There are several limitations to this study such as small sample size, wide range of age of children, unavailability of MRI, considering of all types of epilepsy with different etiologies, selecting of patients in an urban tertiary referral center and collection of data based on parental history.

In conclusion, in evaluation of epileptic children, more concern should be paid to key points such as age of seizure onset, seizure type and developmental status in taking their history and routine brain CT scan is unnecessary and unjustified in all epileptic children and might be considered in evaluation of children with partial seizures, seizure onset in less than one year of age and neurodevelopmental delay.

**Table 1:** Frequency Distribution of Abnormal Developmental Status and Partial Seizure Based on Age of Seizure Onset

**Age of seizure onset** **Data**		**< 1year**	**≥ 1 year**	**Total**	**P.Value**
Seizure type	Generalized	16	81	97	0.02
	Partial	22	31	53	
Mental /developmental status	Normal	27	85	112	0.6
	Abnormal	11	27	38	
Total		38	112	150	

**Table 2:** Comparison of Sex Distribution, Mean Of Age and Seizure Onset Age of Patients Based on CT Scan Result

**Brain CT scan result**		**Normal**	**Abnormal**	**P.value**
**Data**				
Sex	Girl	44	18	
	Boy	67	21	
Age in years (Mean ± SD)		6.8 ± 3.1	5.97 ± 3.2	0.4
Age of seizure onset in years (Mean ± SD)		4.02 ± 1.9	1.17 ± 0.6	0.02

**Table 3:** Comparison of Seizure Onset Age, Developmental Status and Brain CT Scan Results Based On Seizure Type

Type of seizure		Partial	Generalized	Total	P.Value
Data					
Patient se izure onset age	<1 year	22	16	38	0.02
	≥ 1 year	31	81	112	
Mental /developmental status	Normal	31	81	112	0.04
	Abnormal	22	16	38	
CT scan results	Normal	26	85	111	0.02
	Abnormal	27	12	39	
Total		53	97	150	
